# Ethnic Disparity in Mortality Among Ischemic Heart Disease Patients. A-20 Years Outcome Study From Israel

**DOI:** 10.3389/fcvm.2021.661390

**Published:** 2021-06-30

**Authors:** Arsalan Abu-Much, Eyal Nof, Nicola Luigi Bragazzi, Anan Younis, David Hochstein, Arwa Younis, Nir Shlomo, Alexander Fardman, Ilan Goldenberg, Robert Klempfner, Roy Beinart

**Affiliations:** ^1^Leviev Heart Center, Sackler School of Medicine, Tel Aviv University, Tel Aviv, Israel; ^2^Laboratory for Industrial and Applied Mathematics, Department of Mathematics and Statistics, Centre for Disease Modelling, York University, Toronto, ON, Canada; ^3^St George's Hospital Medical School, University of London, London, United Kingdom; ^4^Heart Research Follow-Up Program, University of Rochester Medical Center, Rochester, NY, United States; ^5^Department of Cardiology, Cardiovascular Research Institute Maastricht, Maastricht University Medical Center, Maastricht, Netherlands

**Keywords:** ischemic heart disease, ethnicity, all-cause mortality, disparity, heart disease

## Abstract

**Background:** Long-term morbidity and mortality data among ischemic heart disease (IHD) patients of different ethnicities are conflicting. We sought to determine the independent association of ethnicity and all-cause mortality over two decades of follow-up of Israeli patients.

**Methods:** Our study comprised 15,524 patients including 958 (6%) Arab patients who had been previously enrolled in the Bezafibrate Infarction Prevention (BIP) registry between February 1, 1990, and October 31, 1992, and subsequently followed-up for long-term mortality. We compared clinical characteristics and outcomes of Israeli Arabs and Jews. Propensity score matching (PSM) (1:2 ratios) was used for validation.

**Results:** Arab patients were significantly younger (56 ± 7 years vs. 60 ± 7 years; *p* < 0.001; respectively), and had more cardiovascular disease (CVD) risk factors. Kaplan-Meier survival analysis showed that all-cause mortality was significantly higher among Arab patients (67 vs. 61%; log-rank *p* < 0.001). Multivariate adjusted analysis showed that mortality risk was 49% greater (HR 1.49; 95% CI: 1.37–1.62; *p* < 0.001) among Arabs.

**Conclusions:** Arab ethnicity is independently associated with an increased 20-year all-cause mortality among patients with established IHD.

## Introduction

Despite the latest medical achievements, ischemic heart disease (IHD) continues to be a leading cause of death in some of middle and high-income countries (1). In Israel, IHD is the second leading cause of death (2). Several studies reported ethnic disparities in presentation and outcome of cardiovascular diseases over the world which have been attributed to variability in prevalence of cardiovascular risk factors, differences in screening for primary prevention as well as poor health literacy, health care seeking behaviors and compliance to treatment among specific ethnicities (3, 4).

Jews constitute the majority of Israeli citizens (74.7%) while Arabs are a sizable minority of the whole population (20.8%) (5). Interestingly, genetic studies suggest common genetic features of Arabs and Jews demonstrated by remarkable similarity in Y-chromosome haplotype composition and average frequency (6). Yet, there are major differences seen in some disorders that are typical for each group members (7, 8). National studies have shown conflicting results in the role of ethnicity in cardiovascular diseases (CVD) outcomes. Some showed an increased mortality among Arabs following acute myocardial infarction (9) and greater frequency of non-ischemic cardiomyopathy as well as of sudden cardiac death, while others showed no difference (10, 11). We hypothesized that Israeli Arabs and Jews with established IHD may have diverse characteristics due to differences in their environmental and socioeconomic background. Accordingly, we aimed to evaluate these differences and to assess their impact on long-term all-cause mortality.

## Methods

### Study Population

**Figure 1:**
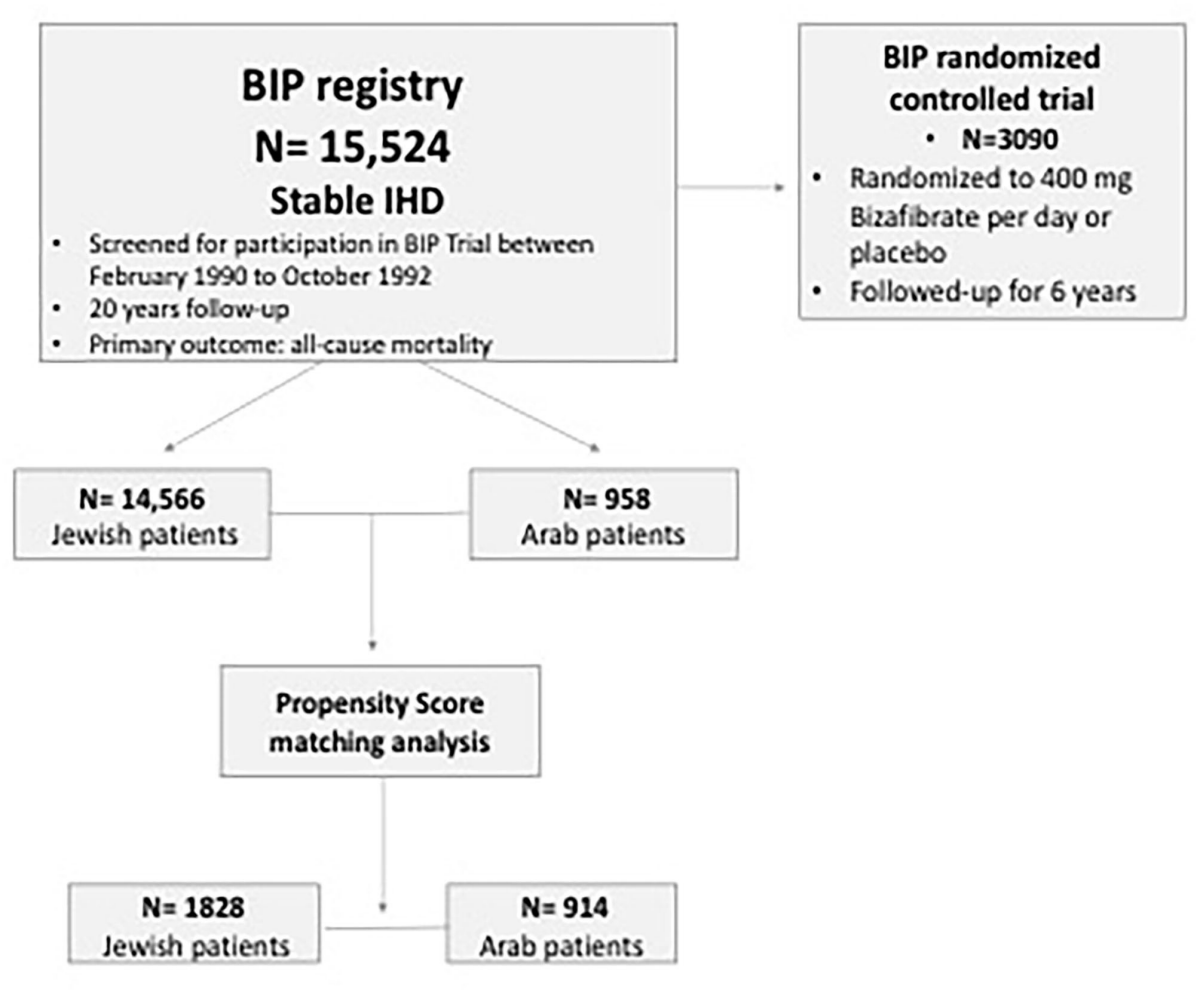
Study flow chart. BIP, Bezafibrate Infarction Prevention; IHD, ischemic heart disease.

The present study population comprised patients who were screened for participation in the prospective multicenter randomized Bezafibrate Infarction Prevention (BIP) trial from February 1990 to October 1992 and enrolled in the BIP Registry. The design and rationale of the BIP Registry and study were published previously (12–14). Briefly, the BIP Registry included 15,524 patients aged 40–74 years with stable IHD. Of these patients, 20% were enrolled in the randomized prospective interventional 6-year BIP study that compared Bezafibrate to placebo (Figure 1). Major exclusion criteria for the BIP study were permanent pacemaker implantation, chronic hepatic or chronic kidney disease (CKD) (GFR < 60 mL/min/1.73 m^2^ or Creatinine >2 mg/dl), peripheral vascular disease, malignant diseases, and type 1 diabetes mellitus.

The study was approved by our institute's internal review board and was performed according to the principles expressed in the Declaration of Helsinki and the ethics policy of the institute and patients signed an informed consent upon their enrollment.

### Definitions and Endpoints

#### Ethnic Groups

Ethnic groups were categorized according to the Statistical Abstract of Israel 2020 (15): Jews and Arabs. Arabs included Muslims (including Circassians), Arab Christians (including Armenians), Arab Druze and others (non-Arab Christians, members of other religions, and not classified by religion).

#### Primary End Point

The primary end point of this study was all-cause mortality.

### Statistical Analysis

Variables were expressed as means ± standard deviation (SD) or median and inter-quartile range (IQR). Categorical data were summarized as numbers and percentages. The demographic, clinical characteristics and laboratory values of patients at baseline according to the pre-specified groups were compared with the use of the *t*-test for continuous variables normally distributed and the Mann-Whitney test for a parametric comparison, the chi-square test was performed to compare categorical variables.

Kaplan–Meier survival analysis was used to estimate survival of subjects among the groups. The subsequent long-term survival probability, was compared using the Log rank test. Univariate and multivariate Cox proportional hazard (PH) regression modeling was used to assess the association between ethnicity and long-term mortality. This was adjusted for different covariates.

In addition, Propensity score matching (1:2 ratio) analysis was constructed from patient's characteristics to control for substantial difference between study groups. Using logistic regression model, we computed conditional probability for each subject to be in the Arab group using multiple covariate and explanatory variables (Supplementary Table 1).

We also performed a sensitivity analysis excluding subjects randomized to the BIP study (*n* = 3,090).

Statistical significance was accepted as two-sided *p* < 0.05. Statistical analysis was performed with IBM SPSS version 20·0 (Chicago, IL, USA) R programming.

## Results

### Clinical Characteristics

Our current study population comprised 15,524 patients aged 59 ± 6 years, of whom 80.7% were males. The vast majority of patients were Jews [14,566 subjects (93.8%)] while only 6.17% (958 subjects) were Arabs. The baseline characteristics of patients by ethnic origin are summarized in Table 1.

**Table 1:** Baseline characteristics of the study population by ethnicity.

	**Arabs**	**Jews**	***p*-value**	**MSD**
*N* (%)	958 (6.17)	14,566 (93.8)	–	–
Age (±SD)	55.88 (±6.82)	60.12 (±6.95)	<0.001	0.615
Gender (%)	831 (86.7)	11,698 (80.3)	<0.001	0.174
eGFR; mL/min (±SD)	86.37 (±14.73)	80.78 (±14.17)	<0.001	0.387
Past MI (%)	737 (77.2)	10,458 (72.0)	<0.001	0.118
Type 2 DM (%)	261 (27.3)	2,722 (18.7)	<0.001	0.204
Hypertension (%)	252 (26.6)	4,925 (33.9)	<0.001	0.161
Past Stroke (%)	13 (1.4)	259 (1.8)	0.411	0.034
COPD (%)	47 (4.9)	410 (2.8)	<0.001	0.109
Current smoker (%)	183 (19.1)	1,585 (10.9)	<0.001	0.232
NYHA-FC ≥ 2 (%)	323 (34.2)	3,956 (27.9)	<0.001	0.136
Total cholesterol; mg/dl (±SD)	222.57 (±42.38)	224.34 (±39.40)	0.182	0.043
Fasting glucose; mg/dl (±SD)	124.55 (±61.21)	113.56 (±44.97)	<0.001	0.205
HDL-c; mg/dl (±SD)	34.84 (±8.40)	38.00 (10.25)	<0.001	0.336
Triglycerides; mg/dl (±SD)	187.64 (±113.39)	155.63 (±87.62)	<0.001	0.316

*MSD, Mean Standardized Difference; SD, Standard Deviation; eGFR, Estimated Glomerular Filtration Rate; MI, Myocardial Infarction; DM, Diabetes Mellitus; COPD, Chronic Obstructive Pulmonary Disease; NYHA-FC, New York Heart Association-Functional Class; HDL-c, High-Density Lipoprotein Cholesterol*.

Notably, Arabs patients were significantly younger than Jews (56 ± 7 years vs. 60 ± 7 years), had higher prevalence of CVD risk factors, higher rates of previous myocardial infarction as well as lower functional status according to New York Heart Association (NYHA). In fact, Arabs were active smokers almost twice the amount compared to Jews (19.1 vs. 10.9%; *p* < 0.001). Arab patients were also found to have higher prevalence of diabetes (27.3 vs. 18.7%; *p* < 0.001), prior myocardial infarction (77.2 vs. 72%; *p* = 0.001), chronic obstructive pulmonary disease (4.9 vs. 2.8%; *p* < 0.001), and suffered more from congestive heart failure (NYHA ≥ 2: 34.2 vs. 27.9%; *p* < 0.001).

### Primary Outcome

**Figure 2:**
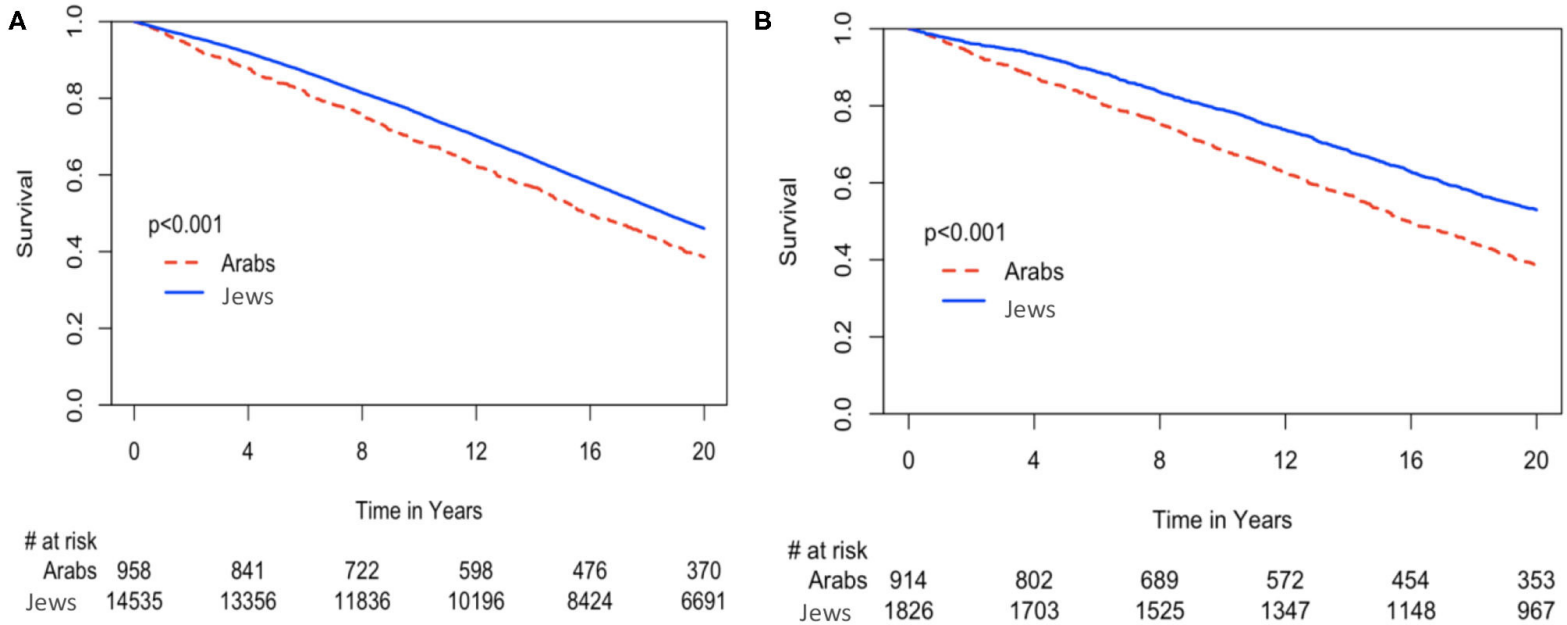
**(A)** Kaplan-Meier of 20-year survival estimates for the entire cohort according to ethnicity. **(B)** Kaplan-Meier of 20-year survival estimates for the propensity score matched cohort according to ethnicity.

Kaplan-Meier survival analysis showed significantly higher all-cause mortality among Arabs throughout a 20-years follow up, reaching mortality rates of 67% (*n* = 631) compared to 62% (*n* = 8,893) (Arabs vs. Jews, log-rank *p* < 0.001) (Figure 2A).

Accordingly, multivariate analysis showed that Arab ethnicity remained a significant cause for total mortality, with a hazard ratio of 1.49 (95% CI: 1.37–1.62; *p* < 0.001). Other significant factors are shown in Supplementary Table 1.

### Propensity Score Matching

Following further adjustments using propensity score matching (Supplementary Table 2), the survival rates of Arabs remained lower compared to Jews (Figure 2B). In order to avoid statistical bias, we further performed a sensitivity analysis excluding patients enrolled into the BIP randomized trial (Figure 1). Similar results were likewise obtained.

### Subgroup Analysis

**Figure 3:**
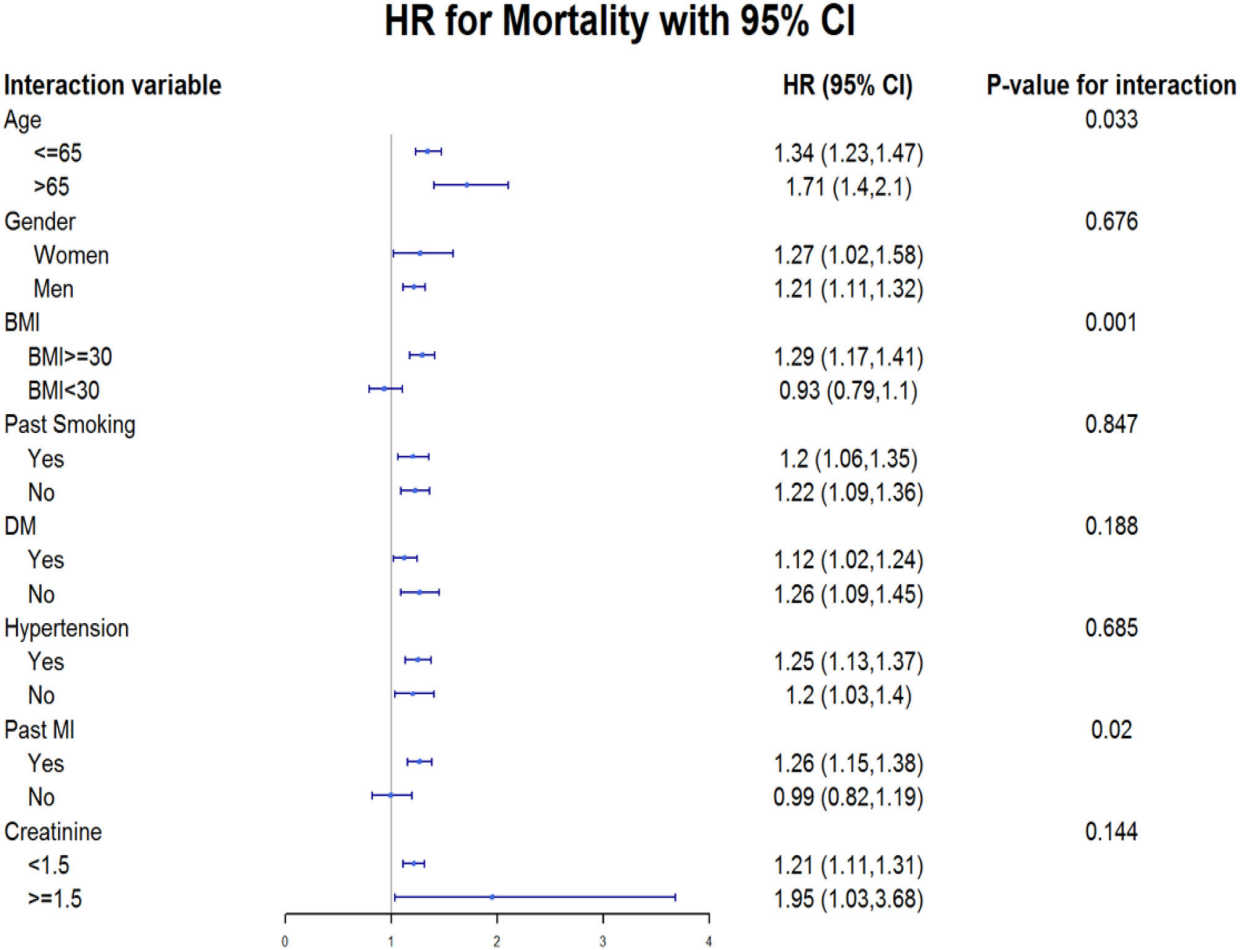
Subgroup analysis predicting the association between 20-year all-cause mortality and Arab ethnicity. HR, Hazard Ratio; CI, Confidence Interval; BMI, Body Mass Index; DM, Diabetes Mellitus; MI, Myocardial Infarction.

We further explored the independent association between Arab ethnicity and long-term mortality in pre-defined subgroups of patients (Figure 3). This analysis showed that HR for mortality was 1.34 (95% CI: 1.23–1.47) in subjects aged equal to or <65 years vs. HR of 1.71 (95% CI: 1.40–2.10) in individuals aged >65 years (*p*-value for interaction 0.033). HR for mortality was 1.29 (95% CI: 1.17–1.41) in people with BMI equal to or >30 kg/m^2^ vs. HR of 0.93 (95% CI: 0.79–1.10) in those with BMI < 30 kg/m^2^ (*p*-value for interaction 0.01). Finally, HR for mortality was 1.26 (95% CI: 1.15–1.38) in those with a past history of MI vs. HR of 0.99 (95% CI: 0.82–1.19) in those without a history of MI (*p*-value for interaction 0.02). Gender (*p*-value for interaction 0.676), smoking status (*p*-value for interaction 0.847), DM (*p*-value for interaction 0.188), hypertension (*p*-value for interaction 0.685) and creatinine (*p*-value for interaction 0.144) did not achieve statistical significance.

## Discussion

To the best of our knowledge, this study entailed the largest Israeli national sample of IHD patients evaluating ethnic disparities and assessing their impact on clinical outcomes throughout a span of time longer than 20 years.

The findings of our study can be summarized as follow:

1. The study identified Arab ethnicity as an independent risk for all-cause mortality among patients with IHD.
2. Survival rates remained lower among Arabs throughout the follow-up period.
3. Arabs with IHD are younger compared to Jews and have higher rates of previous myocardial infarction.
4. Arabs were found to have more CVD risk factors including diabetes, high triglycerides levels, high BMI, low HDL levels, and higher smoking rates.

Despite the genetic affinities between Jews and Arabs, disparities in mortality have been reported. A previous study found that Israeli Arabs had 1.4-times greater all-cause mortality during a relatively short term follow up of 1 year. Death rate was 1.8-times higher due to heart diseases, doubled higher due to diabetes mellitus and 1.7-times greater due to cerebrovascular diseases (16). In contrast to these reports, other studies (10, 11) failed to detect any mortality differences.

For example, Gotsman et al. explored the differences in heart failure patients. Their study included 6,773 patients and found no differences in mortality. They, however, reported that Arab males had higher rates of cardiac-related hospitalizations and death (10). Our study reported on significant higher mortality rates in a much larger patient population. In contrast to previous studies which had a limited follow up of 12–18 months, our study continued for more than two decades. We show here for the first time that the differences in overall mortality persisted throughout the entire follow up period.

### Potential Contributing Factors

#### Diabetes, Obesity, and Metabolic Syndrome

In keeping with previous studies (10), we also report higher rates of classic CVD risk factors including diabetes and obesity in the Arab population. In fact, Arabs are diagnosed with diabetes 10 years earlier compared to Jew (17, 18). This could be attributed to genetic factors, lower socio-economic status, in addition to consumption of unhealthy diet and lower level of physical activity (18). Furthermore, Arabs have higher BMI values especially among women (17, 19) as well as higher prevalence (70%) of metabolic syndrome among obese population (20). Similar findings were also shown in a study of the pediatric population, where obesity and overweight were higher among Arab children across all age groups compared to Jewish children (21). As metabolic syndrome and diabetes are major contributors to all-cause mortality and cardiovascular mortality in the long term (22), the above mentioned could explain the differences in mortality trends demonstrated in our study.

#### Cigarette Smoking

In 2014, the Israeli Ministry of Health reported higher prevalence of smoking among Arab men in Israel (46%), and similar rates persisted over the past decade. In contrast, smoking among Jewish men has declined by 48% over the last 30 years (to ~20% in 2014) (23). Those observations are highlighted by our study, where Arabs were found to be more active smokers than Jews. Despite education, data suggests that Arabs are less likely to quit smoking. It could be that other advanced measures should be taken in the Arab population, addressing cultural complexity (24).

### Coronary Artery Disease

Several previous reports showed that Arabs presented at a younger age with acute myocardial infarction or heart failure (9–11, 25). This is consistent with our data, showing that history of myocardial infarction was more prevalent among Arabs during study enrollment. Thus, suggesting that early onset of coronary artery disease (25) may eventually lead to premature death. Other studies involving the Arab population in other countries reported similar results (25, 26). These data might imply genetic factors as contributing to accelerated atherosclerosis rather than environmental factors (27).

A possible mechanistic explanation for the above findings may be attributed to higher levels of GlycA in Palestinians compared to Israelis living in Jerusalem (21). This is a novel biomarker of systemic inflammation (28) that has been shown to predict cardiovascular diseases and is associated with increased risk for all-cause and cardiovascular mortality (21). Further studies are needed for wider validation of this theory.

### Adherence to Medical Treatment and Advice

Our study and other studies (10) report that both glucose and lipid profiles were consistently higher in Arabs as compared to Jews. This finding might be related to different response to medication, but could also be attributed to lower compliance rates among Arabs. Ethnic disparity in adherence to medication was previously described. Minorities (Asians, Hispanics, Native Americans, and African Americans) were less adherent to heart failure medications even after adjusting for income and drug coverage (29), a factor that might serve as possible explanation for dissimilarities in outcomes. In addition, Arabs participate less in cardiac prevention and rehabilitation programs following acute coronary syndrome, a fact that could further contribute to our findings (30).

### Socioeconomic Status and Access to Medical Therapy

The Arab has a lower income per capita compared to the Jewish population (31) and is generally defined as a “lower socioeconomic status community” in Israel. Interestingly, socioeconomic deprivation has been linked to high incidence of heart failure as well as poor outcomes regardless of cardiovascular risk factors (32). This correlation could be attributed to decreased access to healthcare services, transportation cost, inequalities in treatment and lower affordability of medications (32, 33). This interesting finding was also observed among hypertrophic cardiomyopathy patients (34).

## Limitations

This is a retrospective analysis of a prospective study. Possible unmeasured confounders such as socioeconomic status, may have biased the results; therefore, our results should be interpreted as hypothesis generating. In order to minimize this bias, we performed PSM as well as sensitivity analysis. Despite the many advantages of using long-term data from randomized controlled trials, patients from these trials may do better than real world patients. Thus, our results may not be applicable to elderly (age >80 years) or those with advance renal dysfunction (GFR < 60 mL/min/1.73 m^2^ or Creatinine >2mg/dl) which have been excluded from the beginning. Furthermore, our study doesn't include cardiovascular outcomes and instead, it included only all-cause mortality as the only available long-term outcome in the BIP-study. This fact limits our ability to imply a cardiovascular etiology to patient's outcomes.

## Conclusions

Arab Ethnicity is independently associated with increased 20-year all-cause mortality in patients with stable CAD. This association was consistent even after performing a propensity score matching. Different factors, that included early development of diabetes and obesity, cigarette smoking and premature CAD were highly correlated with the poor outcome. Intensive screening and prevention programs are needed to address those factors early in adulthood.

## Data Availability Statement

The original contributions presented in the study are included in the article/Supplementary Material, further inquiries can be directed to the corresponding author/s.

## Ethics Statement

The studies involving human participants were reviewed and approved by IRB Sheba Center, Tel Aviv University, Tel Aviv, Israel. The patients/participants provided their written informed consent to participate in this study.

## Author Contributions

AA-M and RB conceived the study and drafted the study. NS, EN, and NB analyzed data. EN, AnY, DH, ArY, NS, AF, IG, and RK critically revised the paper. All authors take responsibility for all aspects of the reliability and freedom from bias of the data presented and their discussed interpretation.

## Conflict of Interest

The authors declare that the research was conducted in the absence of any commercial or financial relationships that could be construed as a potential conflict of interest.
