# Supplementary material for: Ethnic Disparity in Mortality Among Ischemic Heart Disease Patients. A-20 Years Outcome Study From Israel
Source: Front Cardiovasc Med. 2021 Jun 30;8:661390. doi: 10.3389/fcvm.2021.661390 (PMC8277917; doi:10.3389/fcvm.2021.661390)
Supplement: Supplementary file 1 [file Data_Sheet_1.docx]

**SUPPLEMENTARY MATERIAL**

**Ethnic Disparity in Mortality Among Ischemic Heart Disease Patients.**

**A-20 Years Outcome Study from Israel**

Corresponding author:

**Arsalan Abu-Much, MD**

E-mail: arsalanabumuch1@gmail.com

Tel: +972546444022 Fax: +97235305905

Address: Leviev Heart Center, Sheba Medical Center, Tel Hashomer

Ramat Gan, Israel 52621

**SUPPLEMENTARY MATERIAL**

**Table S1. Multivariate Cox Analysis Predicting Long-term Mortality Among the Whole Study Population and by Ethnicity.**

|  | The entire cohort | | | Arabs | | | Jews | | |
| --- | --- | --- | --- | --- | --- | --- | --- | --- | --- |
|  | HR | 95.0% CI | P-value | HR | 95.0% CI | P-value | HR | 95.0% CI | P-value |
|  |  |  |  |  |  |  |  |  |  |
| Arab Ethnicity | 1.49 | 1.37-1.62 | < 0.001 | - | - | - | - | - | - |
| Age (per year) | 1.08 | 1.07-1.08 | < 0.001 | 1.07 | 1.05-1.08 | < 0.001 | 1.08 | 1.07-1.08 | < 0.001 |
| Male | 1.07 | 1.01-1.13 | 0.020 | 1.03 | 0.80-1.32 | 0.83 | 1.07 | 1.02-1.14 | 0.012 |
| BMI (per unit) | 1.16 | 1.10-1.22 | < 0.001 | 0.97 | 0.80-1.17 | 0.76 | 1.18 | 1.11-1.25 | < 0.001 |
| Creatinine * | 1.49 | 1.40-1.59 | < 0.001 | 1.41 | 1.15-1.73 | 0.001 | 1.49 | 1.40-1.60 | < 0.001 |
| Total Cholesterol * | 1.51 | 1.31-1.74 | < 0.001 | 2.15 | 1.13-4.10 | 0.020 | 1.48 | 1.28-1.71 | < 0.001 |
| Fasting Glucose * | 0.99 | 0.94-1.05 | 0.80 | 0.91 | 0.75-1.11 | 0.37 | 1.00 | 0.95-1.06 | 0.92 |
| HDL-c ¶ | 1.54 | 1.44-1.64 | < 0.001 | 1.28 | 1.00-1.64 | 0.046 | 1.56 | 1.46-1.66 | < 0.001 |
| LDL-c * | 1.12 | 1.07-1.16 | < 0.001 | 1.07 | 0.90-1.26 | 0.44 | 1.12 | 1.07-1.17 | < 0.001 |
| COPD | 1.02 | 0.97-1.07 | 0.35 | 0.97 | 0.80-1.18 | 0.77 | 1.03 | 0.98-1.08 | 0.33 |
| Type 2 DM | 1.35 | 1.21-1.51 | < 0.001 | 1.03 | 0.72-1.48 | 0.86 | 1.41 | 1.25-1.58 | < 0.001 |
| Hypertension | 1.34 | 1.26-1.43 | < 0.001 | 1.67 | 1.31-2.14 | < 0.001 | 1.31 | 1.23-1.40 | < 0.001 |
| Past MI | 1.12 | 1.08-1.17 | < 0.001 | 1.02 | 0.84-1.22 | 0.86 | 1.13 | 1.08-1.18 | < 0.001 |
| Current Smoker | 1.38 | 1.31-1.44 | < 0.001 | 1.63 | 1.32-2.01 | < 0.001 | 1.36 | 1.30-1.43 | < 0.001 |
| NYHA-FC >2 | 1.32 | 1.21-1.43 | < 0.001 | 1.20 | 0.91-1.59 | 0.19 | 1.33 | 1.23-1.45 | < 0.001 |
| Past Stroke | 1.35 | 1.17-1.55 | < 0.001 | 1.16 | 0.61-2.22 | 0.65 | 1.37 | 1.18-1.58 | < 0.001 |

HR = Hazard Ratio; CI = Confidence Interval; BMI = Body Mass Index; HDL-c = High-density Lipoprotein Cholesterol; LDL-c = Low-density Lipoprotein Cholesterol; COPD = Chronic Obstructive Pulmonary Disease; DM = Diabetes Mellitus; MI = Myocardial Infarction; NYHA-FC = New York Heart Association Functional Class. * Per mg/dL; ¶ High versus low HDL-c.

**Table S2. Baseline Characteristics Following Propensity Score Matching by Ethnicity.**

|  | **Arabs** | **Jews** | **p-value** | **MSD** |
| --- | --- | --- | --- | --- |
| N | 914 | 1828 | - | - |
| Age (± SD) | 56 (± 6.8) | 55.84 (± 7.2) | 0.513 | 0.027 |
| Gender | 792 (86%) | 1607 (88%) | 0.38 | 0.038 |
| eGFR; mL/min (± SD) | 86 (± 14) | 83 (± 14) | < 0.001 | 0.254 |
| Past MI | 705 (77%) | 1419 (77%) | 0.808 | 0.012 |
| Type 2 DM | 238 (26%) | 454 (24%) | 0.524 | 0.028 |
| Hypertension | 246 (26%) | 461 (25%) | 0.362 | 0.039 |
| Past stroke | 12 (1%) | 22 (1%) | 0.951 | 0.010 |
| COPD | 43 (4%) | 84 (4%) | 0.974 | 0.005 |
| Current Smoker | 168 (18%) | 336 (18%) | 1.000 | < 0.001 |
| NYHA-FC > 2 | 315 (34%) | 626 (34%) | 0.943 | 0.005 |
| Total Cholesterol; mg/dl (± SD) | 222.70 (± 42) | 222.51 (± 39) | 0.907 | 0.005 |
| Fasting Glucose; mg/dl (± SD) | 122.57 (± 58) | 121.46 (± 54) | 0.620 | 0.020 |
| HDL-c; mg/dl (± SD) | 34.99 (± 8) | 35.22 (± 9) | 0.534 | 0.026 |
| Triglycerides; mg/dl (± SD) | 186.19 (± 111) | 180.09 (± 108) | 0.168 | 0.056 |

 MSD = Mean Standardized Difference; SD = Standard Deviation; eGFR = Estimated Glomerular Filtration Rate; MI = Myocardial Infarction; DM = Diabetes Mellitus; COPD = Chronic Obstructive Pulmonary Disease; NYHA-FC = New York Heart Association-Functional Class; HDL-c = High-Density Lipoprotein Cholesterol.
